# The clincal characteristics of abdominal migraine and risk factors for developing migraine later in childhood

**DOI:** 10.1186/1129-2377-14-S1-P4

**Published:** 2013-02-21

**Authors:** KH Lee, EY Kim, JH Lee, SK Kim

**Affiliations:** 1 Department of Pediatrics Kangnam Sacred Heart Hospital, Hallym University Medical Center, Korea

## Objective

Abdominal migraine (AM) is an idiopathic recurrent disorder occurring primarily in children. Because their abdominal pain may be so intensive to interfere with their normal activity and AM is recognized as migraine prodrome, it is important to make the exact diagnosis, appropriate managements and close follow-up the symptoms. We therefore analyzed the clinical characteristics of AM and risk factors for developing migraine later.

## Methods

The 923 children with recurrent abdominal pain (RAP) visited our hospital from Jan 2006 to Dec 2010. Among them we retrospectively studied 84 children fulfilled ICHD-II criteria for AM. Through chart review and telephone interview, we evaluated the clinical characteristics of AM and divided them into two groups by developing for migraine (group A) or not (group B). By comparing the groups, we tried to find the risk factors for developing migraine later.

## Results

About 8.9% of patients with RAP were diagnosed as AM. Their mean age was 7.1± 3.0 years (boys, 24, 28.6%; girls, 60, 71.4%). The frequency of abdominal pain was 4.3± 2.4/week and the duration of abdominal pain was about one hour (0.5~2.0). The symptoms associated with abdominal pain were anorexia (n=35, 15.5%), nausea (n=58, 69.0%), vomiting (n=26, 31%), pallor (n=12, 14.3%) and headache (n=64, 76.2%). 27 (32.1%) patients with AM were suffer from migraine 1.7±0.8 years later from onset of AM, and their mean age was 8.3± 3.2 years (boys, 8, 29.6%; girls, 19, 70.4%). When the clinical characteristics were compared to each other between group A (n=27) and group B (n= 57), there were no differences in age, gender, frequency, duration etc. But AM patients with headache significantly developed migraine later (P = 0.003). AM patients who needed drug therapy significantly developed migraine later (P=0.034).

## Conclusion

32.1% of AM patients developed migraine. And the risk factors for developing migraine were headache associated with AM and general impression by physician that need pharmacologic therapy.
